# An Approach to Assessing Patient Safety in Hospitals in Low-Income Countries

**DOI:** 10.1371/journal.pone.0121628

**Published:** 2015-04-20

**Authors:** Robert Lindfield, Abigail Knight, Daniel Bwonya

**Affiliations:** 1 Clinical Research Department, London School of Hygiene and Tropical Medicine, London, United Kingdom; 2 Ophthalmology Department, Ruharo Mission Hospital, Mbarara, Uganda; Queensland University of Technology, AUSTRALIA

## Abstract

**Objective:**

The aim of the study was to assess non-technical aspects of patient safety practices using non-participant observation in different clinical areas.

**Design:**

Qualitative study using non-participant observation and thematic analysis.

**Setting:**

Two eye care units in Uganda.

**Participants:**

Staff members in each hospital.

**Main outcome measures:**

A set of observations of patient safety practices by staff members in clinical areas that were then coded using thematic analysis.

**Results:**

Twenty codes were developed that explained patient safety practices in the hospitals based on the observations. These were grouped into four themes: the team, the environment, patient-centred care and the process. The complexity of patient safety in each hospital was described using narrative reports to support the thematic analysis. Overall both hospitals demonstrated good patient safety practices however areas for improvement were staff-patient communication, the presence and use of protocols and a focus on consistent practice.

**Conclusions:**

This is the first holistic assessment of patient safety practices in a low-income setting. The methods allowed the complexity of patient safety to be understood and explained with areas of concern highlighted. The next step will be to develop a useful and easy to use tool to measure patient safety practices in low-income settings.

## Background

The World Health Organization (WHO) estimates that up to 1 in 10 patients are harmed by adverse incidents in hospitals not directly related to their clinical care at a cost to health economies of approximately $6 billion per annum. The rate and cost of harm are thought to be greater in hospitals in emerging economies [1].

Evidence suggests that up to one in four cataract surgeries in low income countries result in poor visual acuity [2] (compared to less than 1% of surgeries in high income countries [3]). There are many reasons for this and poor patient safety practices, for example; lack of protocols, poor staff-patient communication or poor infection control, are thought to be part of the root cause [4] and need to be addressed systematically to maximise the number of patients achieving an optimal clinical outcome.

Documenting and understanding patient safety practices in hospitals are important first steps in reducing the risk of harm to patients. Different ways of exploring patient safety in hospitals in emerging economies include studying events that demonstrate poor patient safety practices, such as surgical infections [5] or adverse drug reactions [6]. Case note review is often used to quantify and understand contributing factors. It is also possible to focus on a single issue such as hand-hygiene [7], surgical mortality [8] or prescribing and develop interventions to address them based on the findings of research. This is often studied using qualitative, ethnographic approaches. There are practical issues using case-notes to capture patient safety practices. Whilst useful for collecting quantitative markers of patient safety, they do not necessarily capture the context within which the outcome occurred, and adverse events are not always recorded in case notes which may bias the findings [9]. Ethnographic approaches to assessing patient safety have been used widely, particularly participant or non-participant observation, and are recommended by the WHO as a tool to explore patient safety [10]. They can provide an understanding of culture and context and give rich information about the hospital and the staff that work there.

Focusing on a single issue can limit the understanding of other, related patient safety practices [11]. For example, a study observing hand washing practices may identify that there were no protocols for hand washing, missing the wider absence of protocols within the hospital or the limited capacity to produce them or understand their importance or use. A more holistic approach to observing patient safety practices has been used in high income settings, particularly in operating theatres. Non-participant observation in this setting was used to understand the culture and context of patient safety practices in a clinical area, not focusing on a single issue but observing all actions and interactions over a specific time period [12]. However, similar work has not taken place in low income settings.

Uganda, with a population of 38 million [13], has one of the least efficient health systems globally [14]. It has a tiered government health service with a focus on primary care ‘health centres’ feeding into district, regional and national hospitals[15]. However approximately 50% of health outputs are delivered by the private sector, mainly private-not-for-profit organisations who also operate 70% of training institutions. Both private and public health services are affected by a chronic shortage of health workers, particularly in rural areas, with nearly 50% of posts empty in 2011. Quality of care is a priority for government with the latest Health Sector Strategic Plan [15] acknowledging that previous attention on access to services, whilst important, had not delivered the health improvements required and that different approaches are required to deliver high quality care across private and public providers [15].

This study used non-participant observation in different clinical areas in two hospitals in Uganda to assess and document non-technical patient safety practices, and to develop a tool which could subsequently be used to assess change in practices.

## Methods

### Setting

The study was part of a larger study of quality of care and two hospitals in Uganda agreed to take part. One hospital was a dedicated eye hospital and the other was a general hospital with an eye department.

### Ethical Approval

This was a non-participant observation of non-technical practice in hospital. It was part of a larger study that reviewed the impact of mentorship on the quality of cataract surgery in two hospitals in Uganda.

All staff members were informed of the purpose and methods of the study by the lead researcher before the start of the study. Every staff member was made aware that they could request access to the observation notes at any time and that any request to stop the observations would be adhered to immediately. Verbal permission was sought from the lead staff member in each clinical area before commencing each set of observations.

Observations were made in different clinical areas; as such, there were potentially numerous staff members passing through the observation area during the observation period, which made individually signed consent from each staff member impossible.

Verbal consent was given on behalf of all staff members by the lead staff member in charge of each clinical area when observations took place, and by the lead clinician at both hospitals, on behalf of all staff, at the start of the study which followed an in-depth briefing from the research team.

None of the ethical review groups that approved this study stipulated that we should ask for written consent from staff members being observed or required us to document verbal consent to being observed.

Ethical approval was supplied by the London School of Hygiene & Tropical Medicine, the Ugandan National Medical Council, Mbarara University of Science and Technology and both hospitals’ ethical review boards.

### The patient pathway

The patient pathway was mapped in both hospitals. This involved documenting the steps that a patient having cataract surgery (the most common surgical procedure performed) took from when they entered the outpatient department to when they were finally discharged. Important clinical and non-clinical areas in the pathway were identified and documented. Two independent observers (a hospital doctor not employed by either hospital and a social scientist previously employed as a hospital inspector by the government regulator of patient safety in the UK) received training in non-participant observation from the lead researcher. Training involved documenting a repeated set of short observations in a clinical area in one hospital and reviewing accuracy, completeness and approach. Each area identified in the patient pathway was observed at least twice on two separate occasions over a one week period. Each observation was scheduled to last approximately 30 minutes. During the observation period each observer wrote down a minute-by-minute account of the activities and interactions in the area being observed.

Before the observations began each observer wrote a description of the environment being observed. Information recorded included presence/absence of staff, patients or others, interactions between staff and patients, equipment, furniture, lighting, temperature and any other relevant features that put the observations in context. To ensure that the richness of subjective views of the observer was captured, each observer wrote a reflective note about their thoughts and feelings at the end of each observation period. Immediately after each observation the observers transcribed their observations into MS Word. This gave them the opportunity to review their observations and amend their reflective note. Each transcription consisted of an environmental assessment, detailed observations and a reflective note.

To deal with staff apprehension that their competence was being assessed, all staff were told which areas would be observed ahead of time. It was understood that this might increase the chance of a behaviour change when being observed (i.e. the Hawthorne Effect) however it was felt necessary to allay suspicion. Staff were also encouraged to ask the observers to share their notes with them.

### Analysis

A process of thematic analysis was conducted by the observers and lead researcher based on previous work [16]. This was influenced by a systematic review of patient safety incidents that described key themes associated with these incidents[17] e.g. staff-staff communication. A first round of coding attempted to use existing themes from the systematic review to categorise the observations. New themes were developed that captured the data more precisely (e.g. the concept of disruption). Further rounds of coding/recoding followed, with the development of a code book that allowed the lead researcher to review the coding for each observation and, in discussion with the observers, refine the codes. A final code book was produced that was felt to accurately capture the data. The codes were then refined into themes, both existing and new, that combined codes into distinct categories. These categories provided an overview of codes that were related. Again an iterative process was followed to develop the themes. Coding was conducted using MS Word and manual techniques. The quality of the analysis was assessed by the lead researcher who was not involved in the initial coding or thematic analysis.

The next stage of analysis was to compare the codes and themes in each hospital to assess similarities and differences. A final set of codes and themes were agreed that were relevant to both hospitals.

### Narrative

The final stage of analysis was to capture the nuances of the observations using a narrative of each observed area. This told the story of each observation and included the reflective notes and the environmental assessment as well as the coding.

### Absence of evidence

A key part of the reflective note and narrative was to draw attention to absences of key patient safety approaches from the observations in each clinical area. This required knowledge of the important aspects of patient safety that were required in each area [4, 11, 17]. The patient safety gaps were described in the narrative.

## Results

### Patient Pathway

**Fig 1:**
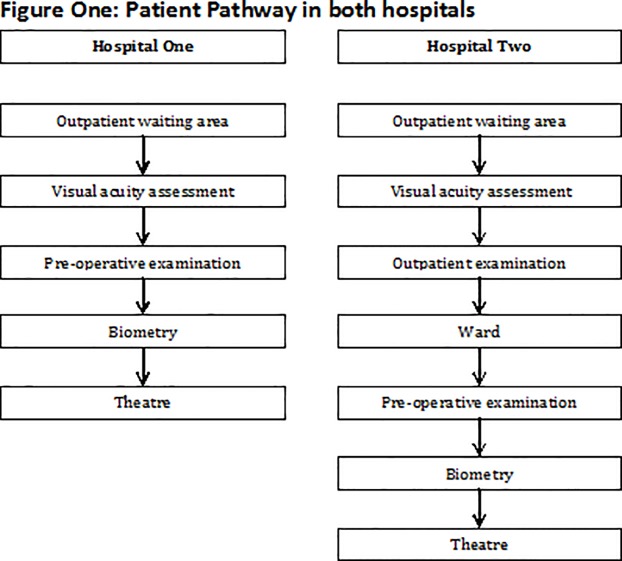
Patient Pathway in both hospitals.

The patient pathways in both hospitals were broadly the same and are described in Fig 1. This led to five sets of observations in one hospital and eight in the second hospital.

### Codes and Themes

The codes, description of codes and themes are described in Table 1.

**Table 1:** List and description of themes and codes.

Theme	Code	Description of the evidence gathered
The team	Staff to staff communication	Staff communicating verbally with each other, either in relation to their roles or more informally. It could include peer to peer communication, more senior staff communicating with more junior staff, and staff communicating between different departments.
	Staff roles	Specific activities performed by various hospital staff cadres, the clarity of their role or demonstration of the staff’s understanding of their role.
	Skills and knowledge	How well the health provider demonstrates, understands and is able to conduct the task at hand
	Staff collaboration	How staff work together in their roles (excluding verbal communication). This includes consultation at same levels or referrals, sharing of information and any observed support.
	Staff availability	Whether staff are available at a specific station at a particular time whenever needed
	Capacity*	The ability of the unit or staff to handle work at hand, e.g. response to unexpected numbers of patients. Over-capacity can be illustrated by staff being unoccupied and under-capacity by long waiting times and related anxiety.
	Supervision	The act of overseeing what other staff are doing. This includes training others in their roles as well as more informal supervision.
Environment	Physical infrastructure	Whether the area/room being observed is appropriate for the task being performed there. Observations include tidiness, arrangement, crowd, ambience and lighting.
	Equipment	Availability and functionality of equipment, including consumables. Includes equipment for documentation, infection control and accident prevention.
	Privacy/confidentiality*	Evidence of the privacy offered to the patient during consultation/procedure.
	Capacity*	The ability of the unit or staff to handle work at hand, e.g. response the OPD gives to unexpected turn up of large number patients. Over-capacity can be illustrated by staff being unoccupied and under-capacity can be illustrated by long waiting times and related anxiety.
	Disruption	Something unrelated to the consultation taking place that happens in the room that may or may not distract the clinician’s attention
Patient-centred care	Staff patient communication	Verbal or non-verbal (e.g. pointing) communication between staff and patients.
	Patient care	Any sign relating to respect and the consideration, or lack of consideration, of the patient as a human being with rights and preferences, including non-verbal communication.
	Privacy/confidentiality*	Privacy offered to the patient during consultation/procedure.
Process	Process	Existence of a standard procedure for performing a task or role. It is evidenced through repeated actions, and a set order of tasks.
	Protocol	Existence of a *documented* outline of a procedure e.g checklist for admitting patient
	Preparedness	Whether the provider is ready in regards to the procedure at hand towards a patient. Examples can include taking time to prepare conditions prior to the patient’s arrival or having to leave part-way through an examination to retrieve something.
	Documentation	Recording patient information. This can include the regularity with which patient information is documented, the system that they have in place for documentation and the checking of patient information during consultations
	Infection control	Any action taken by a health provider either to prevent infection or augment infection transmission. The availability of the necessary consumables and equipment for infection control is coded as ‘equipment’.
	Accident prevention	Practices taken or not taken to avoid accidents and protect patient and staff safety, eg. Safe surgical checklist. It excludes the necessary equipment to perform accident prevention, which is included under the ‘equipment’ code.
	Pathway	Patient’s passage from one service station to another. This includes verbal or written direction, or clarity of patient flow. The pathway is described as clear to the patient or not clear. e.g an observation where a patient fails to locate the next point on services shows that the pathway is not clear.

*Codes to at least two themes.

Twenty codes emerged that described patient safety from the observations which were merged into four unifying themes.

### The Team

Codes relating this theme described how well the team was observed to perform in relation to patient safety. Critical areas within this theme were communication within the team (e.g. non-verbal: “EN squirts saline in P2’s eye and hands syringe to S”, verbal: “EN asks N1 for something”), roles (e.g. “N1 shows in P3 and gives notes to OCO3. OCO3 reads notes”), skills and knowledge (see below), collaboration (e.g. “S checks with TM”), availability (e.g. “There are no nurses in the room”), capacity (e.g. “In the theatre currently there is a surgeon (S), Running Nurse (RN1), an equipment nurse (EN) and a cleaning nurse (CN1) in the adjoining cleaning area.”) and supervision (e.g. “OCO1 consults with D”).

Within the same hospital there were variable examples of team performance. For example: an individual was observed to be extremely familiar with a particular machine, evidenced by his ability to understand and use the machine (“N checking machine. Ensuring wires plugged in”, “N press buttons on machine. Takes battery out. Plugs in to charge”). Another part of this observation that provided evidence of this staff member’s ‘skills and knowledge’ was that he cleaned the equipment between patients. This showed he understood the importance of infection control. The opposite of this was found in another clinical area at the same hospital. Here the staff member showed limited ‘skills and knowledge’ about a procedure (“N points randomly at different figures on the chart”) demonstrating that they did not understand the correct approach.

Frequently communication between staff was good, with lots of evidence of effective communication. A good example of this was in the operating theatre of both hospitals where the scrub nurse was noted to pass the correct surgical instruments to the surgeon without prompting (which also evidenced the ‘skills and knowledge’ code as the nurse demonstrated that they knew their role and understood the procedure).

### The environment

The environment theme captured structural aspects of patient safety such as the availability of space and equipment to provide care that did not compromise patient safety.

The availability and use of equipment in both hospitals was observed to be good with one main exception: the charts used to assess visual acuity in both hospitals were in a poor state of repair and difficult to read. Whilst this might sound a minor point the accurate measurement of visual acuity is the foundation of cataract surgery. If the measurements are inconsistent (as evidenced above) or wrong (as evidenced by “on second testing the patient sees differently”) then the patient may end up having further investigations unnecessarily or, of more concern, may not have the investigations required. Poor visual acuity testing also means that poorly performing surgeons will not be identified.

The aspects of accident prevention that coded to the environment theme revealed hazards that could lead to accidents. For example, electrical wires were strewn across the floor in one hospital and hospital trolleys were broken in the other.

The confidentiality and privacy code of the environment theme required evidence of the availability of space that maintained (or supported the maintenance) of confidentiality and privacy. There were several observations of staff leading patients through rooms where clinical conversations were happening, or screaming children were brought into the operating theatre during surgery during someone else’s operation.

### Patient-centred care

Patient centred care included codes related to observations that showed evidence of care which focused on patients’ needs. This included communication with patients (e.g. “OCO3 answers relative’s questions”), demonstrating care or compassion towards them (e.g. “N2 walks in and helps OCO1xs patient out of room”) and maintaining confidentiality and privacy (e.g. “Patients walks through room holding notes”).

There was very limited evidence of effective communication between staff and patients in both hospitals with patients often being ignored (“nurse does not respond to patient”) in all clinical areas in both hospitals. Despite the lack of evidence related to patient-staff communication there was evidence of patient care in both hospitals “staff helps patient to chair” and “staff helps patient to sit by equipment”.

### The Process

The process theme described codes that showed that systems were in place, either formal (such as documentation) or informal (repeated practice that revealed that the practice was embedded in the delivery of care to patients). A staff member described was observed to repeatedly clean the equipment, which showed that the process of infection control was embedded in his clinical practice. However another staff member was recorded to clean their hands with alcohol once during an observation period suggesting that hand cleaning was not embedded in his practice.

Documentation, which was also a process theme, was variable and patchy. The research team suggested that an important aspect of documentation was that it was consistent, with similar information being documented for similar clinical assessments on every patient. Observations in theatre and of specific procedures such as measurements or interventions, showed consistent documentation but in other locations, for example during an outpatient assessment, documentation was observed to be variable and sometimes non-existent.

Post-operative infection, insertion of the wrong intraocular lens or surgery on the wrong eye can result very poor visual outcomes after cataract surgery. Evidence from the ‘infection control’ code and the ‘accident prevention’ code both provided information about these aspects of care. Infection control was variable with evidence of some good practices, such as cleaning instruments, sterile technique in theatre, and poor practice such as not using gloves, not washing hands. Again this varied within each hospital. As this was not a technical assessment of patient safety, no formal check of sterilisation techniques or effectiveness was performed.

Accident prevention coded to the process and environment themes. The process of accident prevention was evidenced by systems in place that made accidents less likely, for example the eye to be operated on was always marked before the patient went to the operating theatre but neither hospital used the WHO safe surgical checklist or double checked the patient’s name, the eye to be operated or the intraocular lens strength when the patient arrived in the operating theatre.

Very few protocols were observed throughout all clinical areas of both hospitals. Tools like protocols have been shown to improve consistency between staff, something that was observed to be an issue in both hospitals where frequently two staff performing the same task were observed to do so differently.

### Narrative

**Fig 2:**
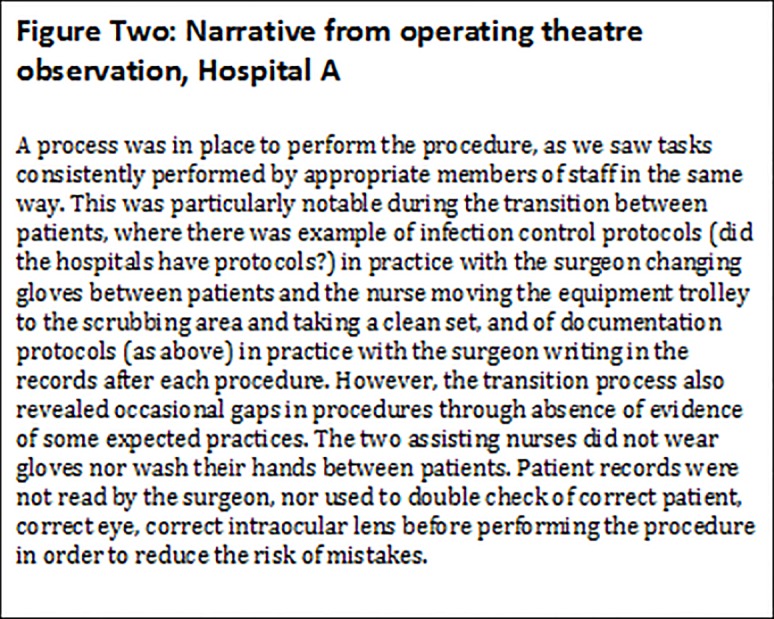
Narrative from operating theatre observation, Hospital A.

**Fig 3:**
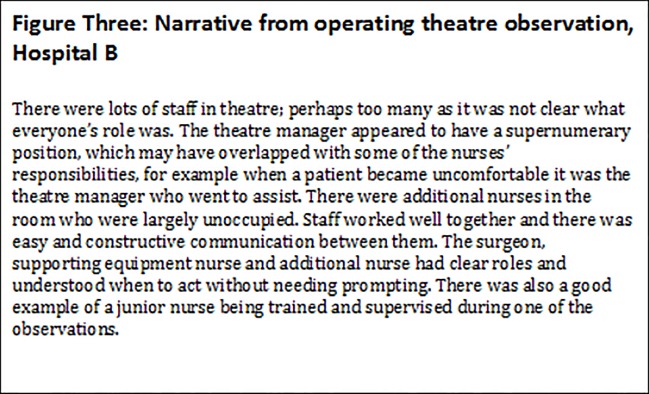
Narrative from operating theatre observation, Hospital B.

The complexity of patient safety practices across different clinical areas in the same hospital, with evidence of good and poor practices, sometimes by the same member of staff, meant that coding alone led to loss of important nuances. Coding also meant that important patient safety issues were given the same weight as potentially less important issues. To deal with these nuances, capture the differences and attempt to draw attention to the most important issues, a narrative was produced describing the evidence from each clinical area. Fig 2 shows part of the narrative from the operating theatre observations in one hospital, showing the importance of evidence of absence of procedures or practices. Fig 3 is part of the narrative from the second hospital illustrating themes relating to capacity, collaboration and roles.

## Discussion

This study assessed the use of non-participant observation to explore patient safety practices in two hospitals in Uganda. Analysis of the observations produced 20 codes which grouped into four themes that described patient safety in these settings. The themes were the team, the environment, patient-centred care and the process. These were then used to produce narrative vignettes that described the situation in different clinical areas including important absences of patient safety practices.

The variation in observed practice was found to be best described using narratives as they provided a means of describing nuances that were not picked up in simple thematic analysis. The narrative allowed an assessment of the central tendency of the themes whilst allowing for variation both within and between staff members and clinical areas.

We found that, overall; both hospitals were safe places for patients. Specific issues consistently arose from the analysis, most commonly about patient-centred care, particularly staff-patient communication, and process, such as lack of protocols, and some poor infection control processes. There was also an issue of consistency as safety practices were not uniform; in the same clinical area there were examples of both good and poor practices. An important action for both hospitals would be to use the narratives to identify evidence of good practice and learn from it.

The codes produced were in this study were similar to those in the systematic review which assessed patient safety errors [17]. Some important differences between the systematic review and our thematic analysis were in our study wider context issues (i.e., political, economic or regulatory factors) were not included as they are difficult to observe. Contextual factors are widely acknowledged to influence patient safety practices [17] although we had no evidence from the observations that the wider context within which the hospital functioned had any impact on the hospital’s capacity to provide a safe environment for patients.

Many of the codes required evidence of a culture of patient safety as they included factors that needed some level of organisation at a hospital-wide level. For example, the presence of protocols, the presence of adequate numbers of trained staff, the availability of infection control measures (e.g. soap, water, towels) would all be evidence that the hospital had considered these important enough to invest in, even if they not had not been specifically introduced to address patient safety.

### Reflexivity

Two of the three researchers were from the UK and their view of patient safety was developed by working in that environment with its cultural norms. These norms might not be relevant in other contexts. For example, the expectation that staff communicate with patients in a specific way may differ in Uganda. However, on discussion with the researcher from Uganda, who did not have a UK perspective, it was felt that these differences, if they existed, were minor.

### Using the process

The coding of observations was relatively straightforward and it is felt that those without a research background could use this approach to explore patient safety practices in their institution. However it must be emphasised that the observations are only part of a complete assessment of patient safety. We learnt little about the technical competence of staff in performing any procedure (i.e. surgery) or whether best practice processes were being followed for more technical aspects of care such as sterilisation of instruments, cleaning or maintaining the sterile environment.

### Limitations

The observations also only took place on two occasions over the course of a week and it is possible that a completely different picture may emerge if the observations took place over a longer period of time. It is also possible that individual staff members behaved differently because they were being observed, particularly as they were fully informed about the observations beforehand. It was felt that if they behaved differently it would most likely be in the direction of what they thought they should be doing. Despite this, some important safety practices, such as hand washing were still not observed. It was not possible to observe all activities taking place during the observation period some significant problems might have been missed. To minimise this risk two independent observers were used and they conducted two sets of observations at different times.

### Conclusion

This is the first holistic assessment of the non-technical aspects of patient safety in a low income hospital setting. It revealed certain issues about the hospitals that could, if used, help them improve patient safety.

As an approach it was straightforward and comprehensive and it is planned to use this data to develop a tool to evaluate interventions to improve patient safety practices in low-income settings.
